# Mammography screening and mortality by risk status in the California teachers study

**DOI:** 10.1186/s12885-021-09071-1

**Published:** 2021-12-18

**Authors:** Hannah Lui Park, Jenny Chang, Vikram Haridass, Sophia S. Wang, Argyrios Ziogas, Hoda Anton-Culver

**Affiliations:** 1 Department of Pathology and Laboratory Medicine, School of Medicine, University of California, Irvine, CA USA; 2 Department of Epidemiology, School of Medicine, University of California, Irvine, CA USA; 3 Department of Medicine, School of Medicine, University of California, Irvine, CA USA; 4 Department of Population Sciences, City of Hope, Duarte, CA USA

**Keywords:** Mammography, Mortality, Breast cancer risk, Cohort study

## Abstract

**Background:**

The debate continues among medical professionals regarding the frequency, starting age, and stopping age for mammography screening. Some experts suggest tailoring recommendations based on individuals’ personal breast cancer risk. Previous studies have not compared the impact of annual versus biennial mammography stratified by age group and risk category. The purpose of this study was to examine the relationship between mammography frequency and mortality by age group and risk category in the California Teachers Study.

**Methods:**

Using data from study questionnaires from 93,438 women between the ages of 40 and 85 and linkages to the California Cancer Registry and other indices, overall and breast cancer-specific mortality by mammography frequency were estimated using multivariable Cox proportional hazards models, stratified by age group and risk category at baseline as determined by the Gail breast cancer risk model.

**Results:**

During the follow-up period of 20 years, overall mortality risk was lower in women who had annual or biennial mammography compared to less frequent or no mammography in all age groups. Annual mammography was associated with lower overall mortality risk compared to biennial mammography among women age 50–85. This difference was especially apparent in women age 60–74, regardless of estimated Gail risk category at baseline. Breast cancer-specific mortality was lower among women who had annual mammography compared to biennial or less frequent mammography among women age 60–74, regardless of their baseline risk.

**Conclusions:**

Our findings suggest that at least biennial mammography is beneficial to most women age 40–85 and that annual mammography is more beneficial than biennial mammography to most women age 50–85 in terms of overall mortality.

**Supplementary Information:**

The online version contains supplementary material available at 10.1186/s12885-021-09071-1.

## Background

Different professional and scientific organizations have different recommendations regarding the frequency, starting age, and stopping age for mammography screening in women. For example, currently the U.S. Preventive Services Task Force (USPSTF) recommends biennial mammography for most women age 50–74 [1], whereas the American Cancer Society (ACS) recommends annual mammography starting at age 45, then biennial screening from age 55 for as long as the woman has a 10-year life-expectancy [2]. Meanwhile, the American College of Radiologists (ACR) [3], American College of Obstetrics and Gynecology (ACOG) [4] and National Comprehensive Cancer Network (NCCN) [5] also each have their own recommendations. Most mention that women should make individualized decisions to start screening earlier or to screen more frequently based on factors including their own personal risk. Recommendations for women age ≥ 75 range from annual mammography (ACR [3] and NCCN [5]) to no recommendation (USPSTF [1]).

Systematic reviews of randomized clinical trials have generally reported that screening in women age ≥ 40 is associated with decreased breast cancer mortality, with a larger magnitude of benefits observed in women age 50–69 compared to 40–49 [6–9]; however, other studies do not agree [10–13]. The concept of an individual’s breast cancer risk playing a role in determining the mammography starting/stopping age or frequency has been a topic of interest, but there is no clear consensus on a recommendation. There is much variation among women and their referring providers’ on their opinions and mammography practices [14–16].

While there have been studies examining the impact of getting mammography compared to not getting mammography on mortality in women with different risk statuses [17, 18], to our knowledge, previous analyses did not compare mortality risk in women who had annual versus biennial mammography stratified by age group and risk category. The purpose of this study was to examine screening mammography patterns among women in the California Teachers Study (CTS), a large, well-characterized prospective cohort, and to examine the relationships between mammography screening frequency, overall mortality, and breast cancer-specific mortality by age group and risk category during a follow-up period of 20 years.

## Material and methods

### Study population

The California Teachers Study (CTS) is a prospective cohort of current or retired female public school teachers and administrators who were members of the California State Teachers Retirement System at the time of study inception in 1995–1996. As previously described [19], 133,477 women joined the CTS and completed a 16-page self-administered baseline questionnaire regarding their demographics, personal and family health history, lifestyle factors, and cancer screening history (including mammography) (https://www.calteachersstudy.org/past-questionnaires).

We sequentially excluded from analyses women who at baseline were residing outside of California (*n* = 8851), had a history of breast cancer at baseline (*n* = 6216), were age < 40 (*n* = 20,321) or > 85 years (*n* = 1833) at baseline, whose follow-up were < 6 months (*n* = 407), those who had missing information regarding mammography use (*n* = 2407), and those whose baseline questionnaires were invalid or who withdrew from the study (*n* = 6). 93,438 women comprised our analytic cohort (Supplemental Table).

### Outcomes

Invasive breast cancer cases (designated by ICD-O-3 site codes 500–509 [excluding morphology codes 9590–9989]) were identified through annual linkage with the California Cancer Registry (CCR), a population-based, statewide cancer registration system that was legally mandated in California since 1988 and shown to be > 95% complete [20].

Deaths were identified via linkage with the State of California mortality files, the Social Security Administration Death Master File and the National Death Index. For overall mortality analyses, follow-up for this analysis was from baseline questionnaire completion until the earliest of the following: date of death due to any cause, move outside of California, or December 31st, 2015. For breast cancer-specific mortality, follow-up was from baseline until the earliest of the following: date of death due to invasive breast cancer, move outside of California, death due to other causes, or December 31st, 2015.

### Predictors

The main independent variable was derived from questions in the baseline questionnaire which asked if the participant had ever had a mammogram (yes/no) and how long it had been since her last mammogram (less than 1 year, 1 to 2 years, 3+ years). These questions served to estimate a woman’s approximate mammography frequency [21, 22]. For the purpose of this analysis, we combined women who never had a mammogram and women who reported their last mammogram was 3+ years ago into one category (“never/less frequent”). Women who reported their last mammogram was 1–2 years ago were categorized as “biennial,” and women who reported their last mammogram was less than 1 year ago were categorized as “annual.”

Age at baseline was categorized into four groups: 40–49, 50–59, 60–74, and 75–85. Other covariates included race/ethnicity, alcohol consumption, body mass index, lifetime strenuous and moderate physical activity, smoking status, menopause status, comorbidities and hormone therapy use. Participants’ comorbid conditions (history of diabetes, heart attack, stroke, or cancer) were collected from the baseline questionnaire and Office of Statewide Health Planning and Development (OSHPD) hospital discharge records from 1991 to 2015 [23]. In addition, we obtained self-reported number of first-degree relatives who had breast cancer. Estimated five-year breast cancer risk was calculated using the online NCI Breast Cancer Risk Assessment Tool (BCRAT) (http://www.cancer.gov/bcrisktool/), which includes age, race/ethnicity, history of breast biopsies, age at menarche, age when the woman gave birth to her first child, and first-degree family history of breast cancer, based on the Gail model [24], for each participant based on their baseline information. Participants in the highest quintile of Gail risk scores were categorized as high risk; the rest were low/average risk.

### Statistical analysis

Chi-square tests were used in bivariate analyses to test for differences in demographic, behavioral characteristics and family history between mammography frequency groups. Overall and breast cancer-specific mortality rates were calculated for each age group. For each age group, time-to-event analysis was performed for the time from baseline to death or death due to breast cancer using Cox proportional hazards models. Participants who were alive at the end of follow-up were censored for overall mortality analysis. Participants who were alive or died from other causes were censored for breast cancer-specific mortality. Hazard ratios with 95% CI by mammography frequency were estimated adjusting for baseline age, race/ethnicity, alcohol intake, BMI, lifetime physical activity, smoking status, menopause and hormone therapy status, history of heart attack, history of stroke, history of diabetes, or history of any cancer. Time-to-event analysis was also performed stratified by high or low/average Gail risk groups.

All *p*-values were two-tailed. Statistical significance was defined as *p* ≤ 0.05. Data were analyzed using SAS and SAS/STAT Version 9.4 (SAS Institute, Cary, NC).

## Results

Baseline characteristics of the study cohort are listed in Table 1 (as percentages of participants by mammography frequency in each age group) and in the Supplemental Table (as numbers and percentages of participants in each age group). Higher mammography frequency was observed in women age 50–74 (69.0% in women age 50–59 and 69.6% in women age 60–74 were in the annual screening group) compared to younger (46.1% in women age 40–49) and older (55.4% in women age 75–85) women (Table 1). Differences in mammography frequency were observed according to all variables tested (Table 1, all *p*-values ≤0.05), most notably by race/ethnicity, menopause/hormone therapy status, breast cancer family history, and Gail risk score. While differences were observed in all age groups, differences for some variables were most evident in women age 40–49. For example, among women age 40–49, 62.7% of women with at least one first-degree family member with breast cancer had annual mammography compared to 44.0% of women with no family history. Similarly, 69.5% of women in the highest quintile of Gail 5-year risk had annual mammography compared to 39.8% in the lowest quintile (Table 1).

**Table 1:** Baseline characteristics of study participants by age group and self-reported mammography frequency (*n* = 93,438)*

	Age at baseline 40–49 (*n* = 31,398)			Age at baseline 50–59 (*n* = 28,342)			Age at baseline 60–74 (*n* = 25,674)			Age at baseline 75–85 (*n* = 8024)		
	Never/ Less frequent (Row%)	Biennial (Row%)	Annual (Row%)	Never/ Less frequent (Row%)	Biennial (Row%)	Annual (Row%)	Never/ Less frequent (Row%)	Biennial (Row%)	Annual (Row%)	Never/ Less frequent (Row%)	Biennial (Row%)	Annual (Row%)
Overall	18.8	35.3	46.1	7.6	22.9	69.0	8.2	22.3	69.6	15.2	29.4	55.4
Mean follow-up time (years)	18.7	18.9	18.8	17.8	17.9	18.0	15.6	16.2	16.7	9.7	11.3	12.0
Race/ethnicity												
White	17.6	35.1	47.3	7.5	22.2	70.3	8.1	21.8	70.1	15.0	29.0	56.0
African American	22.3	38.0	39.7	7.0	29.4	63.6	6.6	27.0	66.5	18.7	35.3	46.0
Hispanic	25.3	37.3	37.4	8.9	29.2	61.9	8.1	26.0	66.0	17.5	27.5	55.0
Asian/Pacific Islander	22.5	36.7	40.7	8.3	27.0	64.6	10.0	27.0	63.0	11.5	41.0	47.4
Other	26.0	33.1	40.9	12.2	26.0	61.8	11.5	25.5	62.9	17.2	32.1	50.7
Number of first-degree relatives diagnosed with breast cancer												
0	19.7	36.3	44.0	7.9	23.8	68.3	8.4	22.7	68.9	15.3	30.1	54.5
1+	10.0	27.3	62.7	5.4	16.8	77.8	6.4	18.4	75.2	11.8	25.6	62.6
Unknown	20.0	35.6	44.4	10.5	25.5	64.1	9.7	27.9	62.4	22.0	27.9	50.1
BMI^a^												
Underweight/normal	17.2	35.2	47.6	6.6	21.9	71.5	7.6	21.2	71.2	15.0	29.2	55.8
Overweight	19.4	35.7	45.0	7.5	23.4	69.1	7.9	22.9	69.2	12.5	30.0	57.6
Obese	23.1	34.8	42.1	10.8	25.3	63.8	9.2	24.0	66.8	16.4	32.6	51.0
Unknown	21.3	38.8	39.9	10.5	26.0	63.5	12.9	23.9	63.2	20.1	26.9	53.0
Lifetime physical activity (hours/week/year)												
≤0.5	17.9	35.9	46.1	8.7	23.8	67.5	9.8	23.2	67.0	18.3	29.0	52.6
0.51–3.99	17.5	35.7	46.8	7.0	23.2	69.8	7.4	22.2	70.4	13.9	29.3	56.8
≥4.0	19.8	34.8	45.3	8.2	22.3	69.5	8.5	21.9	69.6	14.5	29.8	55.7
Menopause/Hormone therapy												
Premenopausal	21.3	35.4	43.3	12.0	26.9	61.1	23.1	38.5	38.5	–	–	–
Postmenopausal - HT Never	22.6	34.8	42.6	21.1	27.8	51.1	17.9	26.4	55.7	24.4	31.7	43.8
Postmenopausal - HT Former	15.6	36.5	47.9	10.8	29.1	60.1	10.1	26.5	63.3	14.1	31.4	54.5
Postmenopausal - HT Current	7.9	35.0	57.1	3.3	20.1	76.6	3.2	19.0	77.8	6.9	25.5	67.6
Other	11.3	35.0	53.6	5.5	21.9	72.7	8.4	23.2	68.4	15.9	29.6	54.5
Alcohol consumption												
None	21.3	34.7	44.0	9.2	23.8	67.0	10.2	23.0	66.8	17.8	29.7	52.5
< 20 g/day	17.3	35.7	47.0	7.0	22.6	70.5	7.0	21.9	71.0	12.9	28.7	58.4
≥20 g/day	16.3	36.3	47.5	6.5	21.3	72.1	7.6	21.6	70.8	13.0	28.8	58.2
Unknown	19.7	31.8	48.5	8.5	25.8	65.8	9.0	22.4	68.5	16.9	31.3	51.9
Estimated 5-year risk (at baseline) by Gail model (%)												
Lowest quintile (0.2–0.9)	24.2	36.0	39.8	9.9	28.0	62.1	10.8	27.1	62.1	22.9	41.7	35.4
Second quintile (1.0–1.2)	15.3	37.2	47.5	8.6	24.9	66.5	11.5	24.8	63.7	20.4	28.8	50.8
Third quintile (1.3–1.5)	10.3	34.2	55.4	7.5	22.5	70.0	8.4	23.9	67.7	20.2	32.4	47.3
Fourth quintile (1.6–1.9)	8.6	29.3	62.1	6.2	21.0	72.7	8.7	22.6	68.6	17.5	30.2	52.4
Highest quintile (2.0–11.4)	6.8	23.6	69.5	4.9	16.3	78.7	7.1	20.6	72.3	12.3	28.2	59.6

*Abbreviations*: *BMI* Body Mass Index, *HT* Hormone therapy

*All *p*-values from chi square tests for association between mammography frequency and characteristics in each age group are ≤0.05

^a^BMI category: Underweight or normal (< 25 kg/m^2^), Overweight (25–29.9 kg/m^2^), Obese (≥30 kg/m^2^)

### Overall mortality

During the 20-year follow-up period there were 20,148 deaths (21.6% of the analytic cohort). Univariate and multivariable analyses showed that overall mortality risk was statistically significantly higher in women in the never/less frequent group compared to the biennial and annual groups among all age groups (Table 2, Never/Less frequent as referent). Further, among women in the 50 years and older age groups, overall mortality risk was statistically significantly lower in those who underwent annual mammography compared to biennial mammography (HR = 0.90, 95% CI 0.81, 0.98, *p* < 0.05 in women age 50–59; HR = 0.87, 95% CI 0.83, 0.92, *p* < .001 in women age 60–74; and HR = 0.91, 95% CI 0.86, 0.96, *p* < 0.001 in women age 75–85) (Table 2, Biennial as referent).

**Table 2:** Overall mortality rates by mammography frequency and age at baseline. Multivariable adjusted hazard ratios for overall mortality risk by mammography frequency, stratified by age group and breast cancer risk at baseline^a^

	Age at baseline							
	40–49 (*n* = 31,398)		50–59 (n = 28,342)		60–74 (*n* = 25,674)		75–85 (*n* = 8024)	
**Univariate Analysis**	Death (N)	rate and 95% CI^b^	Death (N)	rate and 95% CI^b^	Death (N)	rate and 95% CI^b^	Death (N)	rate and 95% CI^b^
Overall mortality								
Never/Less frequent	246	225 (198, 254)	253	658 (581, 743)	941	2877 (2697, 3065)	1122	9513 (8968, 10,080)
Biennial	346	165 (148, 183)	611	524 (484, 567)	2324	2514 (2413, 2618)	2050	7679 (7352, 8017)
Annual	508	187 (171, 203)	1629	460 (438, 482)	6342	2127 (2075, 2180)	3776	7069 (6846, 7297)
**Multivariable analysis (Never/Less frequent as referent)**	HR (95% CI)		HR (95% CI)		HR (95% CI)		HR (95% CI)	
Never/Less frequent	ref		ref		ref		ref	
Biennial	0.70 (0.60, 0.83)***		0.83 (0.72, 0.97)*		0.88 (0.82, 0.95)*		0.80 (0.75, 0.86)***	
Annual	0.76 (0.65, 0.89)***		0.75 (0.65, 0.86)***		0.77 (0.72, 0.83)***		0.73 (0.68, 0.78)***	
**Multivariable analysis (Biennial as referent group)**	HR (95% CI)		HR (95% CI)		HR (95% CI)		HR (95% CI)	
Never/Less frequent	1.42 (1.20, 1.68)***		1.20 (1.03, 1.39)*		1.14 (1.05, 1.23)*		1.25 (1.16, 1.34)***	
Biennial	ref		ref		ref		ref	
Annual	1.09 (0.95, 1.25)		0.90 (0.81, 0.98)*		0.87 (0.83, 0.92)***		0.91 (0.86, 0.96)***	
**Stratified multivariable analysis**^**c**^	HR (95% CI)		HR (95% CI)		HR (95% CI)		HR (95% CI)	
Among low/average risk participants								
Never/Less frequent	1.43 (1.21, 1.70)***		1.19 (1.02, 1.40)*		1.14 (1.03, 1.26)*		1.27 (1.15, 1.41)***	
Biennial	ref		ref		ref		ref	
Annual	1.09 (0.94, 1.25)		0.88 (0.79, 0.97)**		0.87 (0.82, 0.93)***		0.94 (0.87, 1.02)	
Among high risk participants								
Never/Less frequent	1.06 (0.27, 4.20)		1.34 (0.85, 2.09)		1.11 (0.98, 1.26)		1.20 (1.08, 1.35)*	
Biennial	ref		ref		ref		ref	
Annual	0.79 (0.39, 1.58)		1.06 (0.80, 1.40)		0.88 (0.82, 0.95)***		0.88 (0.81, 0.95)***	

*Abbreviations*: *HR* Hazard ratio, *CI* confidence interval, *ref*.: referent group

^a^Multivariable models adjusted for age at baseline, race/ethnicity, alcohol consumption, smoking, BMI, lifetime physical activity, menopause and hormone therapy status, comorbidity conditions including heart attack, stroke, diabetes and other cancer

^b^Overall mortality rate (number of deaths per 100,000 person-years)

^c^ High risk participants were those in highest quintile of estimated baseline breast cancer risk as calculated by Gail model; rest of the participants were low/average risk

*p ≤ 0.05, **p ≤ 0.01, ***p ≤ 0.001

Stratified analysis revealed that the increased overall mortality risk in women in the never/less frequent group compared to the biennial group was observed among women in both risk categories among all age groups but statistically significantly in the low/average Gail risk score category (HR = 1.43, 95% CI 1.21, 1.70, p < 0.001 in women 40–49; HR = 1.19, 95% CI 1.02, 1.40, p < 0.05 in women 50–59; and HR = 1.14, 95% CI 1.03, 1.26, p < 0.05 in women 60–74, and HR = 1.27, 95% CI 1.15, 1.41 in women age 75–85) as well as in women age 75–85 (HR = 1.20, 95% CI 1.08, 1.35, *p* < 0.05) in the high risk category.

The decreased overall mortality risk in women age 50 years and older who had annual versus biennial mammograms remained statistically significant among women age 50–74 in the low/average risk category (HR = 0.88, 95% CI 0.79, 0.97, *p* < 0.01 in women 50–59; HR = 0.87, 95% CI 0.82, 0.93, *p* < 0.001 in women 60–74) and in women 60–85 in the high risk category (HR = 0.88, 95% CI 0.82, 0.95, *p* < 0.001 in women 60–74; HR = 0.88, 95% CI 0.81, 0.95, *p* < 0.001 in women 75–85). Among women age 40–49, there was no difference in overall mortality risk between women who had annual versus biennial mammography in either risk category.

### Breast cancer-specific mortality

Similar trends were observed for breast cancer-specific mortality, with significant mortality risk reduction among women age 60–74 in the annual group compared to the biennial and never/less frequent groups. Stratified analysis revealed that this association was consistent regardless of risk category (HR = 0.63, 95% CI 0.42, 0.93, *p* < 0.05 in low/average risk women; HR = 0.46, 95% CI 0.30, 0.71, *p* < 0.001 in high risk women) (Table 3).

**Table 3:** Breast cancer-specific mortality rates by mammography frequency and age at baseline. Multivariable adjusted hazard ratios for breast cancer-specific mortality risk by mammography frequency, stratified by age group and estimated breast cancer risk at baseline^a^

	Age at baseline							
	40–49 (n = 31,398)		50–59 (n = 28,342)		60–74 (n = 25,674)		75–85 (n = 8024)	
**Univariate analysis**	Death (N)	rate and 95% CI^b^	Death (N)	rate and 95% CI^b^	Death (N)	rate and 95% CI^b^	Death (N)	rate and 95% CI^b^
Breast cancer mortality								
Never/Less frequent	30	27 (19, 39)	17	44 (27, 69)	34	104 (73, 144)	11	93 (49, 162)
Biennial	42	20 (15, 27)	41	35 (26, 47)	73	79 (62, 99)	17	64 (38, 100)
Annual	53	19 (15, 25)	144	41 (34, 48)	133	45 (37, 53)	40	75 (54, 101)
**Multivariable analysis (Never/Less frequent as referent)**	HR (95% CI)		HR (95% CI)		HR (95% CI)		HR (95% CI)	
Never/Less frequent	ref		ref		ref		ref	
Biennial	0.67 (0.42, 1.09)		0.86 (0.48, 1.52)		0.79 (0.52, 1.19)		0.67 (0.31, 1.44)	
Annual	0.65 (0.41, 1.04)		1.03 (0.61, 1.74)		0.44 (0.30, 0.66)***		0.76 (0.38, 1.53)	
**Multivariable analysis (Biennial as referent group)**	HR (95% CI)		HR (95% CI)		HR (95% CI)		HR (95% CI)	
Never/Less frequent	1.48 (0.92, 2.40)		1.17 (0.66, 2.07)		1.27 (0.84, 1.93)		1.50 (0.70, 3.25)	
Biennial	ref		ref		ref		ref	
Annual	0.97 (0.65, 1.45)		1.21 (0.85, 1.71)		0.56 (0.42, 0.75)***		1.15 (0.65, 2.04)	
**Stratified multivariable analysis**^**c**^	HR (95% CI)		HR (95% CI)		HR (95% CI)		HR (95% CI)	
Among low/average risk participants								
Never/Less frequent	1.56 (0.95, 2.55)		1.24 (0.65, 2.34)		0.94 (0.50, 1.75)		1.30 (0.45, 3.74)	
Biennial	ref		ref		ref		ref	
Annual	0.93 (0.61, 1.44)		1.22 (0.82, 1.82)		0.63 (0.42, 0.93)*		1.19 (0.52, 2.73)	
Among high risk participants								
Never/Less frequent	0.62 (0.06, 6.38)		1.13 (0.30, 4.27)		1.63 (0.92, 2.88)		1.74 (0.56, 5.43)	
Biennial	ref		ref		ref		ref	
Annual	0.52 (0.15, 1.78)		0.98 (0.46, 2.09)		0.46 (0.30, 0.71)***		1.21 (0.53, 2.74)	

*Abbreviations*: *HR* Hazard ratio, *CI* confidence interval

^a^ Multivariable models adjusted by age at baseline, race/ethnicity, alcohol consumption, smoking, BMI, lifetime physical activity, menopause and hormone therapy status, comorbidity conditions including heart attack, stroke, diabetes and cancer. Different referent groups were used in the overall model

^b^ Breast cancer-specific mortality rate (number of breast cancer-specific deaths per 100,000 person-years)

^c^ High risk participants were those in highest quintile of estimated baseline breast cancer risk as calculated by Gail model; rest of the participants were low/average risk

*p ≤ 0.05, ***p ≤ 0.001

## Discussion

With the disparate recommendations issued by organizations in the U.S. and elsewhere [25–29] and suggestions for personalized screening [30–32], we sought to examine the relationship between mammography screening frequency and mortality risk with consideration of age and estimated personal breast cancer risk category in the CTS, a large, well-characterized cohort of women with 20 years of follow-up. Since the biggest uncertainty and inconsistency between the recommendations, in addition to starting and stopping ages, pertains to biennial versus annual mammography, our analysis was focused on these two groups.

Despite the USPSTF recommendation during 1995–1996 for all women age ≥ 40 to undergo annual mammography, only 60.3% of study participants in our analytic cohort of 93,438 women reported at baseline that they had their most recent mammogram in the past year. Consistent with other mammography studies, mammography frequency in our cohort was related to some factors classically associated with breast cancer risk, including race/ethnicity, age, and family history [33–37]. That is, women at higher risk for breast cancer (older age, having a first-degree relative with breast cancer) had more frequent mammography compared to women at lower risk, which is generally consistent with the concept of “risk-based screening.” Our finding that higher rates of women who were taking hormone therapy had annual mammography compared to premenopausal women or postmenopausal women who were not taking hormone therapy may be reflective of older age, better compliance with following medical recommendations (since hormone therapy was recommended by the USPSTF and ACOG in 1995–1996 [38]), or better access to health care among these women.

Our results showing that biennial mammography was associated with decreased overall mortality risk compared to never/less frequent mammography among women in all age groups (age 40–85) are consistent with previous studies [7, 8] and may be related to the earlier detection of incident breast cancers. Subsequent analysis will be performed to examine the association for incidence among the 8102 incident breast cancer cases in our cohort. While the differences for women age 40–74 were only statistically significant in the low/average risk group, the trend was similar for women in the high risk groups, suggesting that the lack of statistical significance in these groups may be due to the low sample size of high risk women in the younger age groups. Taken together, our results suggest that biennial mammography is associated with decreased risk of overall mortality for women age 40–85 compared to never/less frequent mammography, regardless of Gail risk category. This result is consistent with the starting age currently recommended by the ACR [3] and NCCN [5], although they recommend annual mammography starting at age 40. ACS [2] and ACOG [4] guidelines state that providers should “offer” mammography to women starting at age 40, while the USPSTF guidelines state that the decision to start mammography before age 50 should be an individual one [1].

Our results that, among women age 60–74, annual mammography was associated with decreased mortality risk compared to biennial mammography, regardless of risk category, are also consistent with current ACR [3] and NCCN [5] recommendations for annual screening for this age group. While our results also indicate that annual mammography was associated with decreased mortality risk among women age 50–59 with low/average risk, there was no association among women in this age group with high baseline risk, potentially due to the smaller sample size in this sub-group. Current recommendations from the ACS [2] and ACOG [4] for women age 50–74 call for biennial or annual mammography, while the USPSTF [1] recommends biennial mammography. While all of these recommendations allude to a shared decision-making process by patients and their health care providers, presumably based on their personal risk and preferences, our results suggest that Gail risk category at baseline would not differentiate between receiving benefit from annual compared to biennial mammography for women age 60–74. This does not, however, preclude the possibility that annual mammography may offer no additional benefit to some women according to risk stratification by other risk models or who meet other criteria.

The USPSTF stated that the current evidence is insufficient to assess the balance of benefits and harms of screening mammography in women aged 75 years or older [1]. However, our results showed that, among women age 75–85, not only was never/less frequent mammography associated with increased overall mortality compared to biennial mammography, annual mammography was also associated with decreased overall mortality compared to biennial mammography. These results are in reasonable alignment with the ACS recommendation for screening as long as the woman has a life expectancy of 10 years [2], as well as the ACOG [4] and NCCN [5] recommendations, which call for consideration of the woman’s current health status, and the ACR [3] recommendations, which do not mention a stopping age.

While we elected to use the modified Gail model (also known as the National Cancer Institute model) because its ease of use and validation in multiple large population databases in the U.S. [39], it would be prudent to do a similar analysis using risk scores calculated according to other validated risk models, including those which consider breast density and genetic profiling of high risk SNPs, since one’s risk score is usually different according to different models [40–45].

Strengths of this study include the large, well-characterized prospective cohort, long follow-up time, high number of incident invasive breast cancer cases and deaths, and inclusion of major breast cancer risk factors including alcohol intake and physical activity in the multivariable analyses. The mortality outcomes were validated by linkage to the CCR and death indices as outlined in the Methods section. Limitations include that the participants were mostly non-Hispanic white, educated, and had health insurance. 87.7% of women age ≥ 40 in our cohort reported having had a mammogram within the past two years (of 1995–1996), compared to the national statistic of 60.9% in 1994 [46]. Thus, the results of this study may not be generalizable to women of different race/ethnicities, socioeconomic status, and access to health care. Also, there are likely other behavioral factors related to breast cancer risk and mortality that we did not account for, like healthful (risk reducing) or harmful (risk increasing) behaviors. Also, we assumed that participants’ patterns of mammography screening did not change after their baseline questionnaire completed in 1995–1996. In 2006, the same mammography questions were asked in Questionnaire 5 (Q5), and there was an 82.8% agreement rate among participants who submitted both Q1 and Q5; thus, this assumption seems reasonable. And while the USPSTF guidelines for breast screening changed in 2009, instead recommending biennial mammograms starting at age 50 for most women, numerous studies have shown that screening practices did not change much after 2009 [14, 47–49]^.^ Further, we performed a sensitivity analysis using this subset of Q1/Q5 participants who had not been diagnosed with breast cancer before Q5, which showed estimates from multivariable models for overall mortality using mammography frequency reported in Q5 were similar to those using mammography frequency reported in Q1 but were not statistically significant due to the smaller number of events (and shorter follow-up time) in this subset (unpublished data).

Lastly, our study did not address potential harms of more frequent mammography, for example, false positive results, over-diagnosis, and decreased quality-adjusted life expectancy. However, these issues were not within the scope of this study, nor were other issues related to policy making. With that said, simulation modeling has shown that women who had higher breast cancer risk had lower rates of false positives and higher gains from screening than lower risk groups [50].

## Conclusions

Our findings suggest that most women age 40–85 would benefit from biennial mammography compared to never/less frequent mammography in terms of overall mortality risk. Further, most women age 50–85 would likely benefit from annual mammography compared to biennial mammography. Among women age 60–74, decreased mortality risk with annual mammography was seen among women in both high and low/average Gail risk categories. Further studies are needed to examine these associations stratified by risk categorizations based on different breast cancer risk models.

## Supplementary Information

**Additional file 1: S1 Table**. Baseline characteristics of 93,438 participants from California Teacher Study by age group.

## Data Availability

The datasets used and/or analyzed during the current study are available upon request via the “For Researchers” tab at https://www.calteachersstudy.org/.

## Abbreviations

CTS: California Teachers Study
USPSTF: U.S. Preventive Services Task Force
ACR: American College of Radiologists
ACOG: American College of Obstetrics and Gynecology
NCCN: National Comprehensive Cancer Network
CCR: California Cancer Registry
OSPHD: Office of Statewide Health Planning and Development
