# Supplementary material for: Mammography screening and mortality by risk status in the California teachers study
Source: BMC Cancer. 2021 Dec 18;21:1341. doi: 10.1186/s12885-021-09071-1 (PMC8684058; doi:10.1186/s12885-021-09071-1)
Supplement: Supplementary file 1 — Additional file 1: S1 Table. Baseline characteristics of 93,438 participants from California Teacher Study by age group. [file 12885_2021_9071_MOESM1_ESM.docx]

| **Supplemental Table 1.** Baseline characteristics of 93,438 participants from California Teacher Study by age group | | | | | | | | |
| --- | --- | --- | --- | --- | --- | --- | --- | --- |
|  |  | Age at baseline 40-49 | Age at baseline 50-59 | Age at baseline 60-74 | | Age at baseline 75-85 | |  |
|  |  | 31398 (33.6) | 28342 (30.3) | 25674 (27.5) | | 8024 (8.6) | |  |
|  |  | N (%) | N (%) | N (%) | | N (%) | |  |
| Race/ethnicity | |  |  |  | |  | |  |
|  | White | 26755 (85.2) | 24962 (88.1) | 22955 (89.4) | | 7379 (92.0) | |  |
|  | African American | 847 (2.7) | 824 (2.9) | 838 (3.3) | | 150 (1.9) | |  |
|  | Hispanic | 1603 (5.1) | 861 (3.0) | 520 (2.0) | | 40 (0.5) | |  |
|  | Asian/Pacific Islander | 1304 (4.2) | 1049 (3.7) | 711 (2.8) | | 78 (1.0) | |  |
|  | Other | 889 (2.8) | 646 (2.3) | 650 (2.5) | | 377 (4.7) | |  |
| Number of first-degree relatives diagnosed with breast cancer | |  |  |  | |  | |  |
|  | 0 | 26851 (85.5) | 23737 (83.8) | 21193 (82.5) | | 6429 (80.1) | |  |
|  | 1+ | 3519 (11.2) | 3706 (13.1) | 3622 (14.1) | | 1154 (14.4) | |  |
|  | Unknown | 1028 (3.3) | 899 (3.2) | 859 (3.3) | | 441 (5.5) | |  |
| BMI^a^ | |  |  |  | |  | |  |
|  | Underweight or normal | 19489 (62.1) | 15820 (55.8) | 13103 (51.0) | | 4083 (50.9) | |  |
|  | Overweight | 6823 (21.7) | 7321 (25.8) | 7388 (28.8) | | 2090 (26.0) | |  |
|  | Obese | 4490 (14.3) | 4439 (15.7) | 3709 (14.4) | | 725 (9.0) | |  |
|  | Unknown | 596 (1.9) | 762 (2.7) | 1474 (5.7) | | 1126 (14.0) | |  |
| Lifetime physical activity (hours/week/year) | |  |  |  | |  | |  |
|  | ≤0.5 | 1918 (6.1) | 3050 (10.8) | 4109 (16.0) | | 2007 (25.0) | |  |
|  | 0.51-3.99 | 15173 (48.3) | 14746 (52.0) | 12469 (48.6) | | 3707 (46.2) | |  |
|  | ≥4.0 | 14307 (45.6) | 10546 (37.2) | 9096 (35.4) | | 2310 (28.8) | |  |
| Menopause/Hormone therapy | |  |  |  | |  | |  |
|  | Premenopausal | 22841 (72.7) | 3734 (13.2) | 13 (0.1) | | 0 (0) | |  |
|  | Postmenopausal - HT Never | 773 (2.5) | 3560 (12.6) | 6070 (23.6) | | 2372 (29.6) | |  |
|  | Postmenopausal - HT Former | 282 (0.9) | 1765 (6.2) | 3640 (14.2) | | 1895 (23.6) | |  |
|  | Postmenopausal - HT Current | 2893 (9.2) | 12827 (45.3) | 13346 (52.0) | | 2508 (31.3) | |  |
|  | Other | 4609 (14.7) | 6456 (22.8) | 2605 (10.1) | | 1249 (15.6) | |  |
| Alcohol consumption | |  |  |  | |  | |  |
|  | None | 9974 (31.8) | 8222 (29.0) | 8048 (31.3) | | 3043 (37.9) | |  |
|  | <20 grams/day | 18184 (57.9) | 16460 (58.1) | 13544 (52.8) | | 3543 (44.2) | |  |
|  | ≥20 grams/day | 2098 (6.7) | 2596 (9.2) | 2763 (10.8) | | 584 (7.3) | |  |
|  | Unknown | 1142 (3.6) | 1064 (3.8) | 1319 (5.1) | | 854 (10.6) | |  |
| Estimated 5-year risk (at baseline) by Gail model (%) | |  |  |  | |  | |  |
|  | Lowest quintile (0.2-0.9) | 16195 (51.6) | 3173 (11.2) | 277 (1.1) | | 48 (0.6) | |  |
|  | Second quintile (1.0-1.2) | 8581 (27.3) | 9760 (34.4) | 1452 (5.7) | | 260(3.2) | |  |
|  | Third quintile (1.3-1.5) | 3461 (11.0) | 7922 (28.0) | 5124 (20.0) | | 786 (9.8) | |  |
|  | Fourth quintile (1.6-1.9) | 2212 (7.0) | 3652 (12.9) | 8707 (33.9) | | 2775 (34.6) | |  |
|  | Highest quintile (2.0-11.4) | 949 (3.0) | 3835 (13.5) | 10114 (39.4) | | 4155 (51.8) | |  |
| Mammography frequency | |  |  |  | |  | |  |
|  | Never/Less frequent | 5838 (18.6) | 2165 (7.6) | 2103 (8.2) | | 1218 (15.2) | |  |
|  | Biennial | 11085 (35.3) | 6499 (22.9) | 5716 (22.3) | | 2357 (29.4) | |  |
|  | Annual | 14475 (46.1) | 19678 (69.4) | 17855 (69.6) | | 4449 (55.4) | |  |
| Abbreviations: BMI, Body Mass Index; HT, Hormone therapy | | | | |  | |  | |
| ^a^BMI category: Underweight or normal (<25 kg/m^2^), Overweight (25-29.9 kg/m^2^), Obese (≥30 kg/m^2^) | | | | | | |  | |
